# Enhanced Near‐Infrared Photoresponse of Inverted Perovskite Solar Cells Through Rational Design of Bulk‐Heterojunction Electron‐Transporting Layers

**DOI:** 10.1002/advs.201901714

**Published:** 2019-09-01

**Authors:** Chih‐I Chen, Shengfan Wu, Yen‐An Lu, Chia‐Chen Lee, Kuo‐Chuan Ho, Zonglong Zhu, Wen‐Chang Chen, Chu‐Chen Chueh

**Affiliations:** ^1^ Department of Chemical Engineering National Taiwan University Taipei 10617 Taiwan; ^2^ Advanced Research Center for Green Materials Science and Technology National Taiwan University Taipei 10617 Taiwan; ^3^ Department of Chemistry City University of Hong Kong Kowloon 999077 Hong Kong

**Keywords:** bulk‐heterojunctions, electron‐transporting layers, inverted perovskite solar cells, NIR photoresponse, nonfullerene acceptors

## Abstract

How to extend the photoresponse of perovskite solar cells (PVSCs) to the region of near‐infrared (NIR)/infrared light has become an appealing research subject in this field since it can better harness the solar irradiation. Herein, the typical fullerene electron‐transporting layer (ETL) of an inverted PVSC is systematically engineered to enhance device's NIR photoresponse. A low bandgap nonfullerene acceptor (NFA) is incorporated into the fullerene ETL aiming to intercept the NIR light passing through the device. However, despite forming type II charge transfer with fullerene, the blended NFA cannot enhance the device's NIR photoresponse, as limited by the poor dissociation of photoexciton induced by NIR light. Fortunately, it can be addressed by adding a p‐type polymer. The ternary bulk‐heterojunction (BHJ) ETL is demonstrated to effectively enhance the device's NIR photoresponse due to the better cascade‐energy‐level alignment and increased hole mobility. By further optimizing the morphology of such a BHJ ETL, the derived PVSC is finally demonstrated to possess a 40% external quantum efficiency at 800 nm with photoresponse extended to the NIR region (to 950 nm), contributing ≈9% of the overall photocurrent. This study unveils an effective and simple approach for enhancing the NIR photoresponse of inverted PVSCs.

## Graphical abstract

**A low bandgap nonfullerene acceptor (NFA)** is incorporated into fullerene electron‐transporting layer (ETL) of an inverted perovskite solar cell aiming to intercept the NIR light passing through the device. However, it cannot enhance the device's NIR photoresponse. Further adding a p‐type polymer effectively enhances the device's NIR photoresponse due to better cascade energy‐level alignment and increased hole mobility.

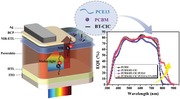

## 1 Introduction

In the past decade, organic–inorganic hybrid perovskite solar cells (PVSCs) have surged a global wave in the photovoltaic community owing to the exceptional optoelectronic properties of the solution‐processable perovskite materials.^1^, ^2^, ^3^, ^4^, ^5^ The state‐of‐the‐art power conversion efficiency (PCE) of PVSC has quickly struck to 24.2% since its first debut in 2009, making them as the most promising photovoltaic materials nowadays.^6^ Hence, myriads of efforts from both academia and industry have been devoted to the development of PVSCs in order to accelerate their progress of commercialization.

Currently, most of the research focuses are aiming to tackle the instability issues of PVSCs and to approach the performance to the Shockley–Queisser limit.^7^, ^8^, ^9^, ^10^ It is worthwhile noting that although the perovskite materials have been proven to possess superior semiconducting properties, their light‐harvesting range is generally limited in the visible‐light region, from 300 to 800 nm, causing great optical loss of solar radiation. To address this issue, low bandgap perovskite materials have been developed through composition engineering to have an extended absorption covering the near‐infrared (NIR)/infrared (IR) light. For example, the bandgap of ABX_3_‐type perovskite materials can be reduced through the replacement of Pb^2+^ cation with Sn^2+^ cation at the B‐site cation or of Cs^+^/MA^+^ cations with FA^+^ cation at the A‐site cation in the lattice. Nevertheless, the resultant performance is either not satisfactory or with a limited extended absorption range.^11^, ^12^, ^13^

Besides composition engineering, device engineering of PVSCs is another feasible method to enhance the NIR/IR photoresponse. For example, the complementary spectra covering the NIR/IR light can be attained through the construction of perovskite‐based tandem cells; however, fabricating a tandem device is quite sophisticated, including the suitable band alignment, optimization of constituent subcells, and proper designs of interconnecting layers/electrodes, which impede its widespread applications.^14^, ^15^, ^16^, ^17^, ^18^ However, the concept of tandem cell could be simplified by directly integrating low bandgap materials into charge‐transporting interlayers (CTLs) in the devices to reduce the spectral and thermalization loss, thus becoming a more viable strategy.

From 2014 to date, such concept has been embodied in single junction PVSCs, for which low bandgap materials are blended into CTLs forming a bulk‐heterojunction (BHJ) structure to absorb the NIR/IR light passing through the perovskite layer, thereby enhancing device's NIR/IR photoresponse.^19^, ^20^, ^21^, ^22^, ^23^, ^24^, ^25^, ^26^, ^27^, ^28^ Notably, to avoid any possible parasitic absorption to the perovskite layer, such hybrid BHJ structure is generally introduced at the top‐side of device. For example, Yang et al. blended PCBM into a hole‐transporting layer (HTL) consisting of a low bandgap polymer to successfully enhance the NIR photoresponse of a conventional PVSC, unveiling the unique characteristic of integrated BHJ/perovskite device structure.^19^ Soon after, Sun et al. further confirmed the importance of the BHJ structure of the derived HTL on enhancing device's NIR photoresponse.^20^ Standing on this basis, Gao et al. highlighted the critical roles of the blending amount of low bandgap materials and processing additives in resultant device performance and NIR photoresponse because of their important influences on the charge mobility and morphology of the derived HTLs.^21^ More recently, Tan et al. first introduced the nonfullerene material into the HTL and similarly manifested the significant role of its blending ratio in enhancing the utilization of NIR.^22^

Such strategy has also been introduced into the inverted PVSCs, even prior to the conventional counterparts, in which the low bandgap materials are blended into the electron‐transporting layers (ETLs) instead.^23^, ^24^, ^25^, ^27^, ^28^ For instance, in 2014, Ding et al. first proposed an integrated perovskite/BHJ solar cell by blending a low bandgap polymer (PDPP3T) into the PCBM ETL, which can exhibit a photoresponse extended to 970 nm.^23^ Soon after, Li et al. similarly introduced a low bandgap n‐type polymer into the PCBM ETL and proposed the concept of parallel‐like tandem solar cells with enhanced NIR photoresponse.^24^ Meanwhile, similar results were reported by Kim et al.; however, they demonstrated that adding an additional n‐type polymer can further facilitate device's NIR photoresponse owing to the more balanced charge mobility in the device.^25^ Despite these discoveries, such integrated PVSC with an enhanced NIR response almost possesses a mediocre PCE of <16%, suggesting the better design of hybrid BHJ ETL is required.

It should be noted that, compared to the performance of conventional configuration, the progress of integrated BHJ/perovskite‐inverted PVSCs is relatively slow. It is mainly limited by the following two reasons: (i) lacking of efficient ETLs in addition to PCBM and (ii) lacking of low bandgap materials with decent electron mobility. In contrast to the vigorous development of HTLs in conventional PVSCs, the ETLs in inverted configuration mainly rely on PCBM. Despite having a high electron mobility, PCBM has a weak light‐harvesting capability owing to its symmetric geometry. Therefore, to develop a BHJ ETL with NIR light absorption necessitates the blending of PCBM with efficient low bandgap materials having decent electron mobility. Recently, the vigorous development of nonfullerene acceptors (NFAs) in organic photovoltaics (OPVs) might address this challenge. On the one hand, the advanced design of NFAs can enable the absorption extended to the NIR region; on the other hand, they generally possess decent electron mobility and compatible energy levels to those of PCBM to allow decent charge transfer and dissociation.^29^, ^30^, ^31^, ^32^

Therefore, in this study, we for the first time, investigate the efficacy of a hybrid NFA/PCBM ETL to enhance the NIR photoresponse of inverted PVSCs. The blended NFA with an absorption extended to the NIR region is aimed to intercept the NIR light passing through the device. Nevertheless, we found such binary ETL could not effectively enhance device's NIR photoresponse despite forming a type II charge transfer between PCBM and NFA. Surprisingly, adding a p‐type polymer into the binary ETL can effectively mediate the charge dissociation so as to enhance device's NIR photoresponse. The importance of the cascade‐energy‐level alignment and morphology of the ternary ETL on derived device's NIR photoresponse as well as PCE is systematically studied. Finally, an inverted PVSC with a PCE of 18.0% is demonstrated, which possesses a 40% external quantum efficiency at 800 nm and the photoresponse is extended to the NIR region (to 950 nm), contributing ≈9% of the overall photocurrent. Our study thus describes an effective and simple approach to enhance the NIR photoresponse of inverted PVSCs by combining the recent tide of NFAs in OPVs.

## 2 Results and Discussion

### 2.1 Binary Bulk‐Heterojunction Electron‐Transporting Layers

**Figure 1:**
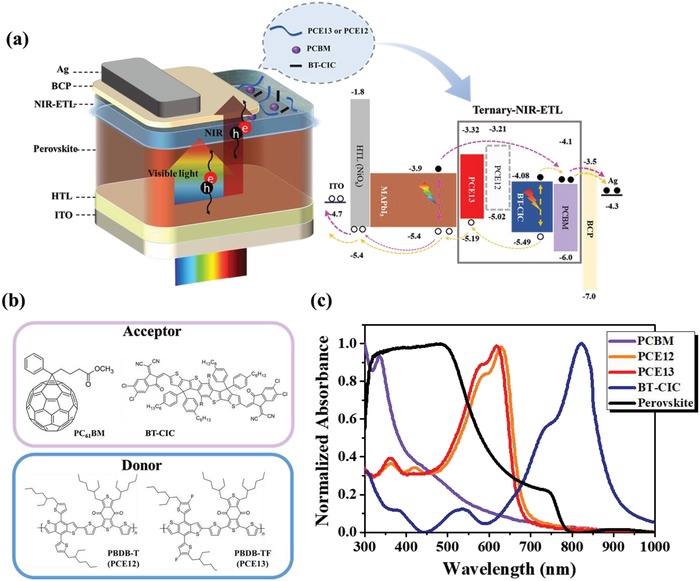
a) Device configuration of inverted perovskite solar cell with BHJ‐electron transporting layer and the energy‐level diagram of the studied device and the proposed mechanism of exciton formation and charge dissociation/transfer. b) The chemical structures of the studied materials including PC_61_BM, BT‐CIC, PBDB‐T (PCE12), and PBDB‐TF (PCE13) and c) their corresponding absorption.

We herein employ a representative NFA, BT‐CIC (**Figure** **1**b, top), as the research object owing to its extended absorption in the NIR region (Figure 1c) and decent charge mobility as reported in the literature.^29^ As portrayed in Figure 1a, the photoexciton of BT‐CIC generated after absorbing the NIR light can form type II charge transfer with PCBM to enable possible charge dissociation. Therefore, using BT‐CIC/PCBM blend as the hybrid ETL in an inverted PVSC is feasible to enhance device's NIR photoresponse, contributing to the resultant photocurrent. To testify this, a binary ETL consisting of BT‐CIC and PCBM is prepared, and the transmittance of the device stack without the top electrode is presented in Figure S1 of the Supporting Information. As shown, compared to the case of using a bare PCBM ETL, such binary ETL indeed enhances the absorption of NIR light of the derived device stack.

**Figure 2:**
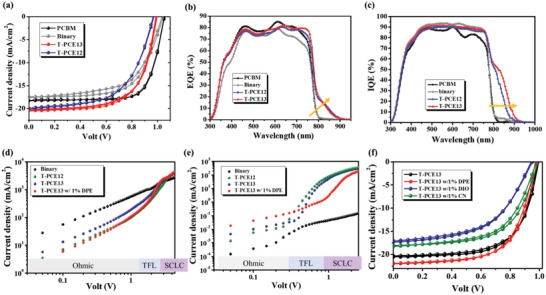
a) The *J*–*V* characteristics, b) EQE, and c) IQE of the studied devices using different ETLs as indicated. SCLC measurements of the d) electron‐dominant device and e) hole‐dominant device based on the studied ETLs. f) The *J*–*V* characteristics of the devices using a T‐PCE13 ETL processed with different additives.

We next examine the photovoltaic performance of these studied devices. Their current density–voltage (*J*–*V*) characteristics measured under AM 1.5 G solar illumination (100 mW cm^−2^) are shown in **Figure** **2**a and the related photovoltaic parameters, including open‐circuit voltage (*V*_oc_), short‐circuit current density (*J*_sc_), and fill factor (FF), are summarized in **Table** **1**. As seen, adding BT‐CIC into the fullerene ETL slightly decreases the overall performance mainly due to the significant drop in FF. It can be attributed to the inferior electron mobility of BT‐CIC to PCBM, highlighting the unique electron‐transporting capability of fullerene in the inverted PVSCs.^33^, ^34^ In addition, such binary ETL fails to convert any NIR photons into the resulting photocurrent as evidenced by the corresponding external quantum efficiency (EQE) spectrum (Figure 2b). However, it is noted that there is a slight increase in the NIR region in the corresponding internal quantum efficiency (IQE) spectrum (Figure 2c). This result suggests that the NIR‐induced photoexciton possesses poor charge dissociation, thus hindering device's NIR photoresponse. Such limitation remains even if we raise the concentration of BT‐CIC in the hybrid ETL as shown in Figure S2 of the Supporting Information. A higher loading of BT‐CIC in the binary ETL even results in a worse EQE curve owing to the decreased electron‐transporting capability.

**Table 1:** The photovoltaic performance of studied PVSCs using different ETLs

Perovskite	ETLs		Scan^a)^	*V*_oc_ [V]	FF [%]	PCE [%]	*J*_sc_ [mA cm^2^]	
							*J*–*V* ^b)^	EQE^c)^
CH_3_NH_3_PbI_3_	PCBM		f	1.05	73	14.1	18.3	19.4
			r	1.05	74	14.3	18.3	
	Binary^d)^		f	1.05	64	11.7	17.4	17.8
			r	1.04	60	10.9	17.6	
	T‐PCE12		f	0.97	59	11.3	19.9	20.2
			r	0.97	58	11.2	20.0	
		T‐PCE13 w/o additive	f	0.99	65	13.1	20.5	20.4
			r	0.99	64	13.0	20.3	
		T‐PCE13 w/1% DPE	f	0.97	64	13.6	22.0	21.1
			r	0.98	64	13.8	21.9	
(FAPbI_3_)_0.85_ (MAPbBr_3_)_0.15_	PCBM		r	1.15	79	18.9	20.7	20.0
	T‐PCE13 w/1% DPE		r	1.14	70	18.0	22.5	21.8

^a)^ f: measured under forward scan; r: measured under reverse scan

^b)^ The current density derived from *J*–*V* curve when applied voltage is 0 V

^c)^ The integrated current density from 300 to 1000 nm

^d)^ BT‐CIC:PCBM = 4:16 mg mL^−1^.

### 2.2 Ternary Bulk‐Heterojunction Electron‐Transporting Layers

Such results are quite confusing since BT‐CIC should be able to form type II charge transfer with PCBM to enable the charge dissociation (Figure 1a). Excluding the absorption disability, one of the important reasons only could be the charge‐transporting problem. PCBM is a well‐known electron‐transporting material. However, to enable efficient charge dissociation, proper hole‐transporting property of the BHJ structure is required, which can ensure the hole transport toward perovskite. We hence speculate that this limitation might be restrained by the poor hole‐transporting property of BT‐CIC, impeding efficient hole transfer from the binary ETL to perovskite. Therefore, we next introduce p‐type polymers into this binary ETL to constitute a ternary ETL aiming to improve its overall hole mobility. We first employ PBDB‐T (PCE12), which has been widely used in OPVs to realize high efficiency, to prepare a ternary ETL (referred as T‐PCE12 ETL).^35^, ^36^ Intriguingly, as shown in Figure 2b,c, the device using this ternary ETL exhibits an obviously elevated EQE and IQE in the NIR region (>800 nm). This result clearly confirms the culprit of the limited NIR photoresponse is the poor hole mobility of BT‐CIC. However, despite the improved NIR photoresponse, the overall performance of the device using such hybrid ETL is much inferior to the performance of the control device using a bare PCBM ETL, mainly due to the loss in *V*_oc_ and FF (Table 1).

To ameliorate this deficiency, we envisage that a better energy‐level alignment between the hybrid ETL and perovskite layer is required. As portrayed in Figure 1a, PCE12 possesses a low‐lying highest‐occupied molecular orbital (HOMO) level, which not only imposes a high barrier for hole transfer to perovskite but also limits the resultant *V*_oc_ of the derived device. Therefore, a p‐type polymer with a deeper‐lying HOMO level can enable the derived ternary ETL to possess a better cascade‐energy‐level alignment to compensate the loss in *V*_oc_ and FF; also, it will be preferred if it possesses a higher hole mobility than PCE12.

Based on this rationale, we next replace PCE12 with PBDB‐TF (PCE13) (Figure 1b) to prepare the ternary ETL (referred as T‐PCE13 ETL). As reported, PCE13 possesses a deeper‐lying HOMO level than PCE12 (Figure 1a) and a better charge transporting capability.^37^, ^38^, ^39^ The T‐PCE13 ETL indeed conspicuously promotes the *V*_oc_ and FF of the derived device as expected, resulting in a more comparable PCE (13.0%) to the value (14.3%) of control device (Table 1). Meanwhile, it preserves device's NIR photoresponse as indicated in its EQE spectrum (Figure 2b).

The above results clearly disclose the importance of the cascade‐energy‐level alignment of the hybrid ETL, which greatly influences the charge dissociation and hole transfer at the BHJ/perovskite interface. As shown in Figure S1 of the Supporting Information, the binary ETL actually captures more NIR light than the ternary ETL. Figure S3 of the Supporting Information presents the simulated optical field in the device using a PCBM, binary, and T‐PCE13 ETL, respectively. As seen, by adding BT‐CIC into the ETL, the intensity of NIR light indeed increases in the top side of the device regardless of the addition of p‐type polymer; however, the majority of the light is still trapped inside perovskite layer, resulting in weak intensity of NIR light at the top side near the ETLs. This explains the conspicuous disparity between EQE (Figure 2b) and IQE (Figure 2c) spectra as observed. Besides, this result also manifests that the BT‐CIC surely absorbs the NIR light but might not contribute to the resulting photocurrent due to the severe charge recombination. Note that, as indicated in the IQE spectra, the device using a T‐PCE13 ETL possesses the most enhanced NIR photoresponse among the studied devices, which again confirms the importance of the cascade‐energy‐level design of the hybrid ETL.

To better ascertain this phenomenon, the charge mobilities of the studied binary and ternary ETLs using the space‐charge‐limited current (SCLC) method are measured. The details of device fabrication for the SCLC measurements are described in the Supporting Information. The *J*–*V* characteristics of the electron‐dominant devices and hole‐dominant devices are displayed in Figure 2d,e, respectively. Basically, the SCLC follows the well‐known Mott–Gurney equation and the charge mobility can be derived from the slope of *J*–*V* ^2^ curve in the SCLC region. As summarized in Table S1 of the Supporting Information, for the binary ETL system, the hole mobility (9.39 × 10^−5^ m^2^ V^−1^ s^−1^) is extremely deviated from the electron mobility (1.08 × 10^−2^ m^2^ V^−1^ s^−1^), as limited by the poor p‐type characteristic of BT‐CIC. Such imbalanced charge mobility causes the inefficient dissociation of NIR‐induced photoexciton and the poor hole transport to the perovskite/ETL interface. However, the hole mobility of the hybrid ETL can be significantly increased after adding p‐type polymer, leading to a more balanced electron and hole mobilities. Notably, the increase in hole mobility does not adversely impact the electron mobility despite the reduced concentration of PCBM.^40^, ^41^ It thus suggests that the well cascade‐energy‐level alignment of the hybrid ETL facilitates the electron transport as well.

### 2.3 Morphology Optimization of Bulk‐Heterojunction Electron‐Transporting Layers

In OPVs, processing solvent additives have been reported to have critical influences on the BHJ morphology, which greatly affects the resulting charge dissociation/transport. Besides, the choice of additive tightly depends on the BHJ systems.^42^, ^43^, ^44^, ^45^, ^46^ In this regard, we anticipate that the hybrid BHJ ETL might be able to further improve by using proper processing solvent additives, and three common additives including DPE, DIO, and CN are herein investigated. Among which, DIO has been known to selectively dissolve PCBM aggregation while CN is considered as a good solvent for polymers, and DPE is generally the theta‐solvent of polymers.

**Figure 3:**
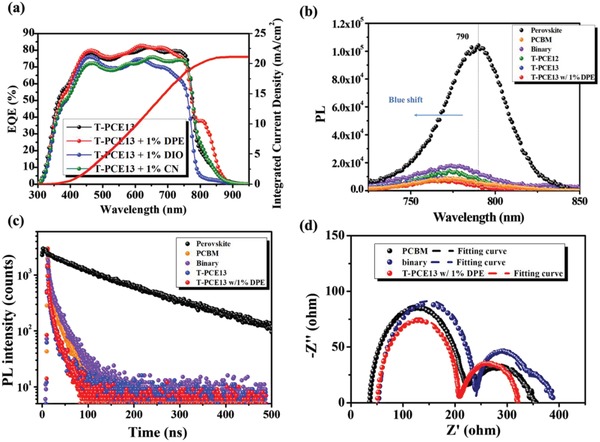
a) EQE spectra of the device using a T‐PCE13 ETLs processed with or without additives, and the integrated current density for the device using a T‐PCE13 w/1% DPE ETL. b) PL spectra and c) TRPL spectra of the studied devices using different ETLs. d) The Nyquist plot of the devices using a PCBM ETL, a binary ETL (BT‐CIC:PCBM = 8:12 mg mL^−1^), and a T‐PCE13 w/1% DPE ETL.

The *J*–*V* characteristics of the PVSCs using a T‐PCE13 ETLs processed with 1% DPE, 1% DIO, and 1% CN (referred as T‐PCE13 w/1% DPE ETL, T‐PCE13 w/1% DIO ETL, and T‐PCE13 w/1% CN ETL, respectively) are presented in Figure 2f and the relevant photovoltaic parameters are summarized in Table S2 of the Supporting Information. Interestingly, the processing additives indeed have a great influence on the resulting performance of derived PVSCs. First, we note that the DIO additive dramatically deteriorates the device performance. It is important to point out that the loading of PCBM in our ternary BHJ ETL is much higher than the general cases of BHJ layers in OPVs. Therefore, the DIO additive seems to incur serious phase separation in the prepared ETL, which even is visible to the naked eyes. It thus drags the device performance down and also suppresses the dissociation of NIR‐induced photoexciton as shown in **Figure** **3**a (EQE) and Figure S4 (IQE, Supporting Information). The CN additive, slightly better than DIO, can maintain device's NIR photoresponse benefitting from the less severe phase separation; however, it seems to hamper the charge transfer across the perovskite/ETL interface, as inferred from the lower EQE between 300 and 800 nm, leading to a reduced overall PCE.

Surprisingly, the DPE additive can largely enhance device's NIR photoresponse. As presented in Figure 3a, the EQE at 800 nm is increased from 20% to ≈40%; meanwhile, the overall PCE is slightly improved from 13.0% to 13.8% (Figure 2f; Figure S5 (Supporting Information), and Table 1). These results indicate this hybrid ETL can intrigue efficient dissociation of NIR‐induced photoexciton while facilitating the charge transfer across the perovskite/ETL interface that can be inferred from its enhanced charge mobility measured by SCLC method (Table S1, Supporting Information). To better analyze the enhanced NIR photoresponse, Figure S6a of the Supporting Information compares the EQE difference and absorbance difference between the devices using a bare PCBM‐ETL and using a T‐PCE13 w/1% DPE ETL. As it can be clearly seen, the enhanced NIR photoresponse originates from the absorption of BT‐CIC, which contributes a photocurrent density of 1.93 mA cm^−2^, representing ≈9% of the overall photocurrent density. The EQE and IQE spectra of this top‐performing device are also compared in Figure S6b of the Supporting Information. Despite the largely enhanced photoresponse in the NIR region, the discrepancy between EQE and IQE spectra in the NIR region is relatively large compared to the region of visible light (300 to 800 nm). It is apparently attributed to its unavoidable optical loss in the device as discussed earlier.

In order to better understand the charge transfer efficiency at the perovskite/ETL interfaces, the interfacial transfer circumstances are measured using photoluminescence (PL) and time‐resolved PL (TRPL). The PL and TRPL spectra of a bare perovskite film and bilayer perovskite/ETL films are measured by exciting the sample from the glass side with a 550 nm wavelength light. As shown in Figure 3b, all the studied hybrid ETLs cause conspicuous quenching of perovskite's emission, suggesting charge transfer occurring at the corresponding interfaces. Moreover, the trend in the degree of PL quenching of the bilayer samples follows the trend of SCLC mobilities of the ETLs (Table S1, Supporting Information). The binary ETL yields the lowest degree of PL quenching due to its lowest SCLC mobilities whereas the T‐PCE13 w/1% DPE ETL delivers the highest degree of PL quenching as a result of its highest SCLC mobility. Interestingly, a gradual blue shift of the emission peak from 775 to 767 nm is observed when the ETL is gradually changed from PCBM to T‐PCE13 w/1% DPE as shown in Figure S7 of the Supporting Information. This result suggests that the introduced BT‐CIC or PCE12/13 could passivate the perovskite interface due to the constituent electron‐rich moiety, such as halogen atoms and π‐conjugated Lewis‐base.^47^, ^48^

Their corresponding TRPL results are presented in Figure 3c and the corresponding PL lifetimes estimated using a biexponential fitting are summarized in Table S3 of the Supporting Information. As shown, the bilayer perovskite/ETL films possess a much more reduced PL lifetime compared to the bare perovskite film, suggesting the existence of charge transfer between them. Besides, the TRPL results of these bilayer films coincide with the trend observed in PL quenching. Similarly, the binary ETL yields the longest PL lifetime in the studied bilayer films, suggesting its poorest charge transfer at the corresponding interface. Except for the binary ETL, the average PL lifetime is gradually shortened from 20.5 to 15.8 ns when the ETL is changed from PCBM to T‐PCE13 w/1% DPE, being consistent with the trend of SCLC mobilities of the ETLs.

**Figure 4:**
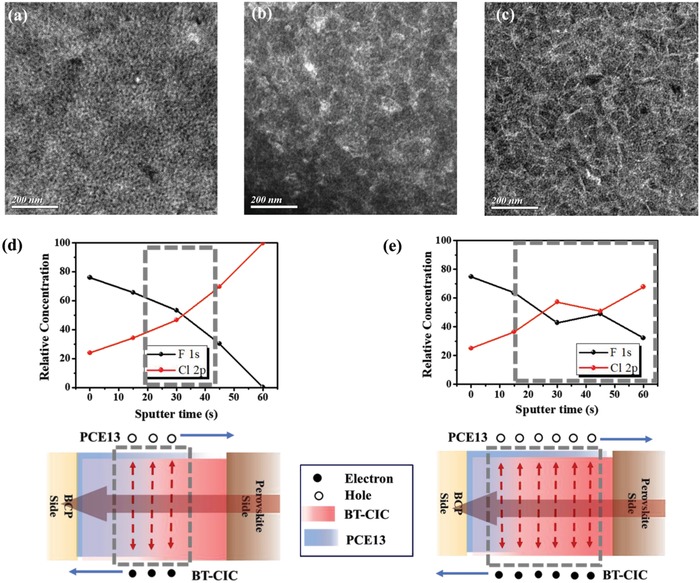
The surface TEM images of a) the binary ETL, b) T‐PCE13 ETL, and c) T‐PCE13 ETL processed with 1% DPE. Normalized XPS element intensity of F atom (related to PCE13) and Cl atom (related to BT‐CIC) for the relatively vertical concentration and associated illustration for distribution of components and dissociation of NIR‐excitons mechanism for d) T‐PCE13 ETL and e) T‐PCE13 w/1% DPE ETL.

As previously mentioned, the BHJ morphology of the hybrid layer plays a pivotal role in its resulting charge dissociation/transport.^42^, ^43^, ^44^, ^45^, ^46^ Hence, it can be envisioned that the different charge transfer efficiencies and charge transport properties observed in these studied hybrid ETLs are strongly correlated with the BHJ morphology. To clarify this issue, the surface morphologies of the binary ETL, the T‐PCE13 ETL, and the T‐PCE13 w/1% DPE ETL are examined using transmission electron microscope (TEM). As presented in **Figure** **4**a–c, all these films possess very distinct surface morphology and texture. Particularly, DPE additive can transform the homogeneous morphology of T‐PCE13 ETL into fibrillary network associated with nanoscale phase separation. Such interpenetrating network is generally believed to be favorable for enhanced charge transport and dissociation, confirming its best performance among the studied hybrid ETLs.

We next investigate the element depth‐profile of the T‐PCE13 ELT and the T‐PCE13 w/1% DPE ETL through X‐ray photoelectron spectroscopy (XPS) measurement. In order to better elucidate the influence of additives, the T‐PCE13 w/1% DIO ETL is also surveyed for a fair comparison. Figure S8 of the Supporting Information presents the XPS element intensity of F atom and Cl atom of the studied ETLs. Note that the intensity of F atom represents the distribution of PCE13 while that of Cl atom represents the distribution of BT‐CIC since only these compounds have these assigned atoms. In order to relatively compare their vertical concentration distribution, the relative concentration is calculated and presented in Figure 4d,e. As seen, PCE13 tends to accumulate near the surface of derived ETL while BT‐CIC is rather even in the vertical distribution for all the cases. It might be due to the better affinity of PCE13 toward surrounding atmosphere during film evolution.^49^, ^50^, ^51^, ^52^ However, the excessive PCE13 near the electrode would hamper the electron transport at the corresponding interface and impede the electron collection efficiency. This might be the main reason for the dropped *V*_oc_ and FF of the device using hybrid ETL as compared to the device using a bare PCBM ETL.

As deduced from the employed ETLs and their different performance, the situation of BHJ region with a D/A ratio equal to ≈1 (gray‐dashed frame) seems to play a crucial role in the performance of the studied hybrid ETLs. As shown in Figure 4d, for the T‐PCE13 ETL, such region mainly exists in the central region and the D/A ratio starts to become extreme at both ends. This means that efficient dissociation of NIR‐induced photoexciton mainly happens in the central region of the hybrid ETL. Meanwhile, the low content of PCE13 at the perovskite side is not optimized for hole transfer to perovskite and its high concentration at the BCP side will hamper the electron transfer to the collecting electrode. However, such uneven distribution is relieved in the T‐PCE13 w/1% DPE ETL as presented in Figure 4e, wherein the BHJ region with a D/A ratio equal to ≈1 is more extended. It clearly explains why the T‐PCE13 w/1% DPE ETL can deliver the best performance among the studied hybrid ETLs. Notably, in contrast to DPE, DIO additive seems to result in severe phase separation between PCE13 and BT‐CIC as portrayed in Figure S9 of the Supporting Information. It thus largely hinders the charge dissociation/transport in its derived ETL, leading to the poor performance as observed. These intriguing results clearly highlight the importance of the BHJ morphology of the hybrid ETLs on resulting performance. Besides, our results suggest that a better design of hybrid ETLs with NIR photoresponse can be realized by careful control of the gradient of the components.

Electrochemical impedance spectroscopy (EIS) was then conducted to probe the charge transfer circumstance in the device. Presented in Figure 3d is the EIS measurement of the devices using a bare PCBM ETL, a binary ETL, and a T‐PCE13 w/1% DPE ETL. The equivalent circuits used for fitting were presented in Figure S10 of the Supporting Information, wherein, in high‐frequency region, *R*_s_ is the series resistance coupled with no capacitance, *R*_bulk_ represents the resistance of transporting in bulk perovskite layer and is closely related to the properties of CTLs except the quality of perovskite itself, and*C*_g_ is the geometric capacitance accounting for the dielectric property of the perovskite layer, while, in low‐frequency region, *C*_2(3)_ states for the ionic accumulation capacitance in dark and the charge accumulation capacitance in light condition and *R*_inter1(2)_ represent the resistance of interfacial recombination.^53^, ^54^ The fitting results are summarized in Table S4 of the Supporting Information and are congruent with the device performance. Note that different from the PCBM and T‐PCE13 w/1% DPE ETLs, the binary ETL obviously displayed two partially overlapped arcs in low‐frequency region, which usually happens when the overall charge transfer rate toward electrodes are not equal or serious charge accumulation exists at associated interfaces.^53^, ^55^ Since all these devices used the same NiO*_x_* HTL, it thus reveals the unbalanced charge transfer of the binary ETL. The poor hole transport across the perovskite/binary ETL interface possibly lowers the driving force for electron transfer toward silver electrode, thereby retarding the charge transport rate. Therefore, we accordingly used a three‐RC equivalent circuit (Figure S10b, Supporting Information) to fit the Nyquist plot of the binary ETL while a two‐RC equivalent circuit (Figure S10a, Supporting Information) was used to fit the curves of PCBM and T‐PCE13 w/1% DPE ETLs.

As seen in Table S4 of the Supporting Information, the device using a binary ETL possesses the highest *R*_s_ of 56.74 Ω and the lowest *R*_inter2_ of 81.45 Ω, suggesting its poorest charge‐transporting capability and the most severe interfacial charge recombination as limited by its low hole mobility. Whereas, the device using a T‐PCE13 w/1% DPE ETL possesses a higher *R*_s_ of 54.71 Ω and a slightly lower *R*_inter1_ of 119.2 Ω, affirming its superior performance than the binary ETL. It should be noted that the EIS results also reflect the slightly inferior photovoltaic performance of the T‐PCE13 w/1% DPE ETL to PCBM ETL. Regardless, T‐PCE13 w/1% DPE ETL possesses a clearly enhanced interfacial charge transport, as evidenced by its lowest *R*_bulk_ of 151.5 Ω. As mentioned, the transporting in bulk perovskite layer not only depends on its film quality but also correlates with the properties of adjacent CTLs. These results thus manifested the positive role p‐type polymer in enhancing interfacial charge transport. Notably, although the PL, TRPL, and *R*_bulk_ results suggest the enhanced interfacial charge transport across the perovskite/T‐PCE13 w/1% DPE ETL interface, the excess PCE13 accumulated at the corresponding interface (as indicated in the XPS results) might impede the electron transfer to incur undesired charge recombination. Consequently, it results in the lower *R*_inter1_ of the T‐PCE13 w/1% DPE ETL than that (139.6 Ω) of PCBM, verifying the importance of vertical element distribution gradient as proposed.

Presented in Figure S11 of the Supporting Information was the stability test of our fabricated devices. As shown in Figure S11a of the Supporting Information, the device using the T‐PCE13 w/1% DPE ETL possessed a comparable ambient stability to the control device using a PCBM ETL, for which the devices were stored in an ambient environment (room temperature with a relative humidity of 60% ± 5%) under dark without encapsulation. This result reveals decent thermodynamic stability of the T‐PCE13 w/1% DPE ETL regardless of its BHJ design, which might be benefitted from the small blending amounts of polymer and NFA. Moreover, we have also tracked the stability of the NIR photoresponse of our fabricated device, wherein three aspects were discussed: (1) the EQE value at 800 nm, (2) the integrated current density above 800 nm, and (3) the contribution of the integrated current density above 800 nm to the overall current density, and the fabricated devices were stored in a glove box or in the ambient environment (room temperature and a relative humidity of 60% ± 5%) under dark without encapsulation. As seen, whether the devices were stored in a glove box or in the ambient environment, the device's NIR photoresponse was well preserved, showing decent stability of the designed T‐PCE13 w/1% DPE ETL (Figure S11b–d, Supporting Information).

### 2.4 General Applicability of Bulk‐Heterojunction Electron‐Transporting Layers

**Figure 5:**
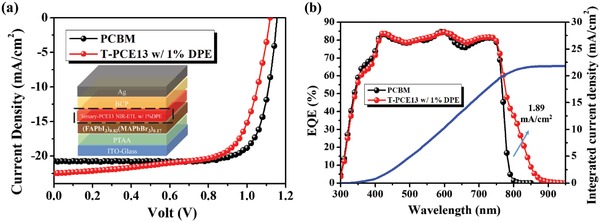
The a) *J*–*V* curves and b) EQE spectra of the (FAPbI_3_)_0.85_(MAPbBr_3_)_0.15_ devices using a bare PCBM ETL and the optimized T‐PCE13 ETL. Inset in (a) is the device configuration and the integrated current density for the device using the optimized T‐PCE13 ETL is also presented in (b).

Finally, we explore the general applicability of our optimized hybrid ETL in another perovskite system and device configuration. A more prevailing perovskite system, (FAPbI_3_)_0.85_(MAPbBr_3_)_0.15_, is thus employed and the NiO*_x_* HTL is replaced by doped poly(bis(4‐phenyl)(2,4,6‐trimethylphenyl)amine) (PTAA) with the purpose of providing higher efficiency.^56^, ^57^, ^58^, ^59^ **Figure** **5**a presents the *J*–*V* characteristics of the fabricated device measured under AM 1.5G solar illumination (100 mW cm^−2^) along with its detail device structure and its corresponding EQE spectrum displayed in Figure 5b. As seen, the T‐PCE13 w/1% DPE ETL indeed enables the high NIR photoresponse of its derived PVSC, wherein the EQE at 800 nm similarly reaches ≈40%. Moreover, the enhanced NIR photoresponse contributes a photocurrent density of 1.89 mA cm^−2^, similarly representing ≈9% of the overall photocurrent density. Combined with the high *V*_oc_ of 1.14 V and decent FF of 70%, an inverted PVSC with a high PCE of 18.0% is finally demonstrated (Table 1). Although this performance is still slightly inferior to the performance (18.9%) of the control device using a PCBM ETL, it is the highest PCE reported for the inverted PVSCs with enhanced NIR photoresponse and with an EQE value over 40% at 800 nm to the best of our knowledge.^25^ Besides, this result also proves the efficacy and well general applicability of our designed ETLs in enhancing the NIR photoresponse of inverted PVSCs.

## 3 Conclusion

In conclusion, we manifest that a BHJ ETL consisting of a mere blend of low bandgap NFAs and PCBM could not effectively convert the NIR light into photocurrent in its derived inverted PVSC. Despite forming type II charge transfer with fullerene, the poor hole mobility of NFAs results in severe charge recombination and limits the generation of NIR photocurrent. However, such dilemma can be resolved by adding a proper p‐type polymer. Owing to the better cascade‐energy‐level alignment and the increased hole mobility, the ternary BHL ETL can significantly enhance derived device's NIR photoresponse. By further optimizing the morphology of the ternary ETL, perovskite composition, and device structure, we finally demonstrate an efficient (PCE: 18.0%) inverted PVSC with enhanced NIR photoresponse, whose external quantum efficiency at 800 nm can reach 40% and extends to the NIR region (to 950 nm), contributing ≈9% of the overall photocurrent density. Our study unveils an effective design strategy of ETL to enhance the NIR photoresponse of its derived inverted PVSC. Despite this success, we note there are some remaining issues of our designed BHJ ETL that need to be solved, such as the accumulation of excess p‐type polymer at the perovskite/ETL interface and the high‐lying HOMO level of the p‐type polymer we used. Hence, based on our results, we anticipate more efficient ETLs with NIR photoresponse can be realized by (i) better selection of NFA with ambipolar charge‐transporting property, (ii) usage of even more high‐performance p‐type polymer, and (iii) careful control of the gradient of the components in the hybrid BHJ ETL.

## 4 Experimental Section

*Materials*: Commercial materials were used as received without further purifications. DIO, CN, and DPE were purchased from Sigma‐Aldrich. PbI_2_ powder (>99.99%) and nickel(II) 2,4‐pentanedionate (95%) were purchased from Alfa Aesar. MAI powder, PC_61_BM (99.5%), and BCP (>99.5%) were obtained from UniRegion. PBDB‐T, PBDB‐TF, and BT‐CIC were purchased from 1‐materials.

*Solution Preparation*: The precursor solution of NiO*_x_* was prepared by dissolving nickel(II) 2,4‐pentanedionate in ethanol with a concentration of 25.7 mg mL^−1^ and with the addition of 10 µL HCl. The PTAA HTL was prepared by dissolving in toluene with a concentration of 10 mg mL^−1^, and then 1 wt% F4‐TCNQ was doped into the PTAA solution. Note that the as‐prepared solution needs to be stirred and heated at 65 °C for 10 h before spin‐coating. CH_3_NH_3_PbI_3_ was prepared by dissolving methylammonium iodide (MAI) and lead (II) iodide (PbI_2_) at a molar ratio of 1:1 into a mixed solvent of DMF:DMSO = 4:1 (v/v) with a concentration of 1 m. (FAPbI_3_)_0.85_(MAPbBr_3_)_0.15_ was prepared by mixing FAI (1 m), PbI_2_ (1.1 m), MABr (0.2 m), and PbBr_2_ (0.2 m) in a mixed solvent of DMF:DMSO = 4:1 (v/v). PC_61_BM was dissolved in DMSO with a concentration of 20 mg mL^−1^. The precursor solution of the binary ETLs was prepared by blending BT‐CIC with PCBM at a ratio of 1:4 or 2:3 (w/w) with a total concentration of 20 mg mL^−1^, while the precursor solutions of T‐PCE12(13) ETL were prepared by replacing part of PC_61_BM with PCE12(13) and the overall weight ratio of p‐type polymer:BT‐CIC:PC_61_BM is 1:1:3 with a total concentration of 20 mg mL^−1^.

*Device Fabrication*: ITO‐glasses were etched and cleaned by DI water, acetone, and isopropanol, followed by the ozone treatment for 35 min. The NiO*_x_* HTLs were spin‐coated onto the ITO‐glasses at 5000 rpm for 10 s and then annealed at 325 °C for 40 min in air. The doped PTAA HTLs were spin‐coated onto the ITO‐glasses at 4000 rpm for 30 s and then annealed at 150 °C for 10 min in glove box. The CH_3_NH_3_PbI_3_ films were spin‐coated onto the NiO*_x_* layer in glove box by a three‐step process: 3000 rpm for 10 s, 4500 rpm for 7 s, and 5500 rpm for 30 s. Sequentially, 400 µL toluene was treated at the end of the second process, followed by annealing at 100 °C for 10 min. (FAPbI_3_)_0.85_(MAPbBr_3_)_0.15_ films were spin‐coated onto the doped PTAA layer at 5000 rpm for 30 s. During the spin‐coating process, 150 µL of chlorobenzene used as antisolvent was dropped onto the center of film at 16 s before the end of spin‐coating, followed by annealing at 100 °C for 30 min. The PCBM ETLs were spin‐coated onto the perovskite films at 2000 rpm for 30 s while the binary/ternary ETLs were spin‐coated at 2000 rpm for 90 s. The BCP layer was spin‐coated at 6000 rpm for 7 s upon the deposited ETLs. Finally, a 100 nm Ag electrode was evaporated under high vacuum (<4 × 10^−6^ Torr). The effective illumination area was defined as 0.1 cm^2^ through shadow mask.

*Characterization*: The UV–vis spectrum was recorded using a Hitachi U‐4100 UV–vis spectrophotometer. Optical simulation was obtained through the transfer matrix formalism, while the parameter needed wavelength complex index of refraction was derived from ellipsometry. The static‐PL emission spectrum was measured by Horiba Fluorolog‐3 spectrometer system. *J*–*V* curves were measured under AM1.5G (100 mW cm^−2^) illumination by an SS‐F5‐3A simulator, Enlitech, recorded with a computer‐controlled Keithley 2400 source. Devices for SCLC mobility measurement were fabricated in ITO/NiO*_x_*/ETLs/MoO_3_/Ag (hole‐dominant device) and ITO/ZnO/ETLs/BCP/Ag (electron‐dominant device) scanning from 0 to 4.5 V under dark. The EQE and IQE spectrum was performed with QE‐R, Enlitech Co., Ltd, AM1.5G reference spectrum and corrected by a single crystal Si photovoltaic cell. TRPL spectrum was recorded with an FLS980 spectrofluorometer (Edinburgh). XPS was conducted through electron spectroscopy for chemical analysis aided with scanning X‐ray monochromator (Al Anode) and 5KV argon ion gun (ULVAC‐PHI PHI 5000 Versaprobe II). TEM was measured with FEI Tecnai G2 T20. EIS analysis was conducted with Autolab potentiostat/galvanostat (PGSTAT 30, Autolab, Eco‐Chemie, Utrecht, the Netherlands) under an illumination of 100 mW cm^−2^ with 0.8 V bias, from 10^7^ to 0.1 Hz.

## Conflict of Interest

The authors declare no conflict of interest.

## Supporting information

Supplementary
