# Supplementary material for: Enhanced Near‐Infrared Photoresponse of Inverted Perovskite Solar Cells Through Rational Design of Bulk‐Heterojunction Electron‐Transporting Layers
Source: Adv Sci (Weinh). 2019 Sep 1;6(21):1901714. doi: 10.1002/advs.201901714 (PMC6839634; doi:10.1002/advs.201901714)
Supplement: Supplementary file 1 — Supplementary [file ADVS-6-1901714-s001.pdf]

Copyright WILEY-VCH Verlag GmbH & Co. KGaA, 69469 Weinheim, Germany, 2019.

ADVANCED SCIENCE
Open Access

Supporting Information

for *Adv. Sci.,* DOI: 10.1002/advs.201901714

Enhanced Near-Infrared Photoresponse of Inverted Perovskite Solar Cells Through Rational Design of Bulk-Heterojunction Electron-Transporting Layers

Chih-I Chen, Shengfan Wu, Yen-An Lu, Chia-Chen Lee, Kuo-Chuan Ho, Zonglong Zhu, Wen-Chang Chen, and Chu-Chen Chueh**

Copyright WILEY-VCH Verlag GmbH & Co. KGaA, 69469 Weinheim, Germany, 2019.

Supporting Information

Enhanced Near-Infrared Photo-response of Inverted Perovskite Solar Cells Through Rational Design of Bulk-Heterojunction Electron-Transporting Layers

Chih-I Chen,[a,b] *Shengfan Wu,*[c] *Yen-An Lu,*[a,b] *Chia-Chen Lee,*[a] *Kuo-Chuan Ho,*[a,b] *Zonglong Zhu,*[c,*] *Wen-Chang Chen,*[a,b] *and Chu-Chen Chueh*[a,b,*]

[a] C.-I. C., Y.-A. Lu, C.-C. Lee, Prof. K.-C. Ho, Prof. W.-C. Chen, and Prof. C.-C. Chueh
Department of Chemical Engineering, National Taiwan University, Taipei 10617, Taiwan

[b] C.-I. C., Y.-A. Lu, Prof. K.-C. Ho, Prof. W.-C. Chen, and Prof. C.-C. Chueh Advanced Research Center for Green Materials Science and Technology, National Taiwan University, Taipei 10617, Taiwan

[c] S. Wu and Prof. Z. Zhu
Department of Chemistry, City University of Hong Kong, Kowloon 999077, Hong Kong

*Corresponding author. E-mail: zonglzhu@cityu.edu.hk; cchueh@ntu.edu.tw

Keywords: NIR photo-response; inverted perovskite solar cell; non-fullerene acceptor; electron-transporting layer; bulk-heterojunction

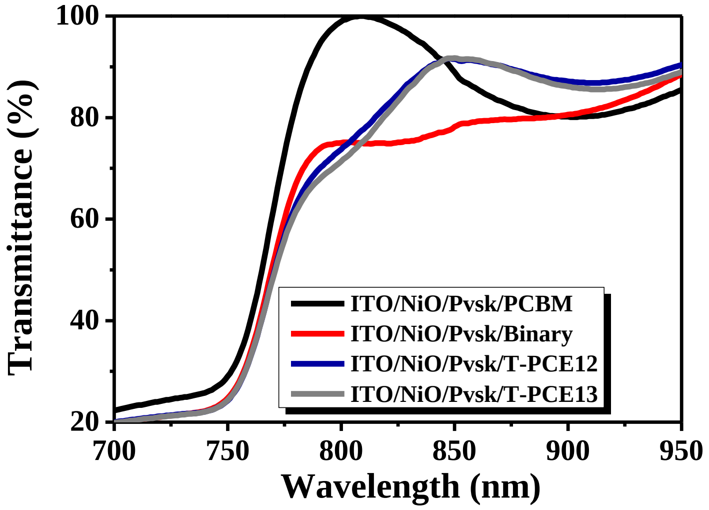

Figure S1. The transmittance spectra of the studied samples as indicated. The binary ETL consists of BT-CIC and PCBM in a weight ratio of 1:4 while The ternary ETLs (T-PCE12 and T-PCE13) consist of polymers, BT-CIC, and PCBM in a weight ratio of 1:1:3.

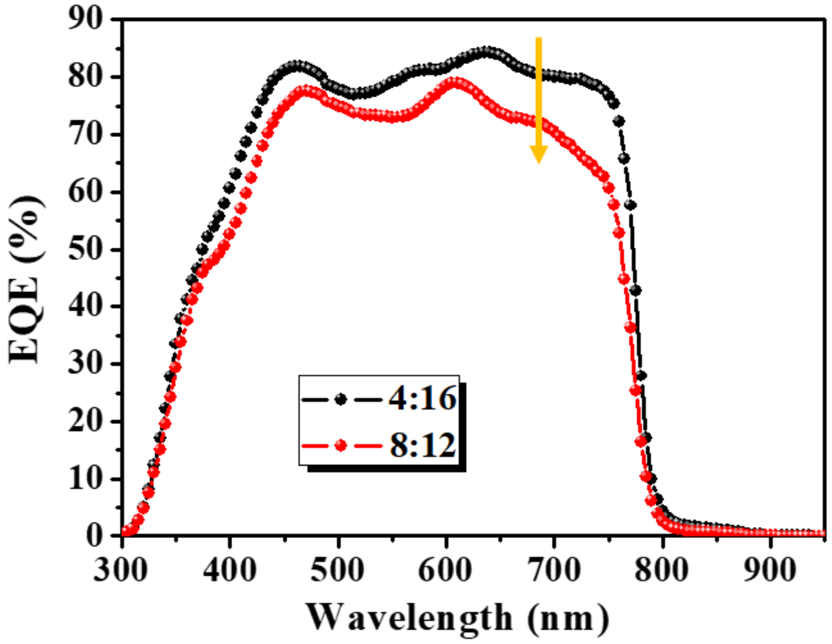

Figure S2. The EQE spectra of the devices using a binary ETL with varied weight ratios of BT-CIC to PCBM under a fixed total concentration of 20 mg/ml.

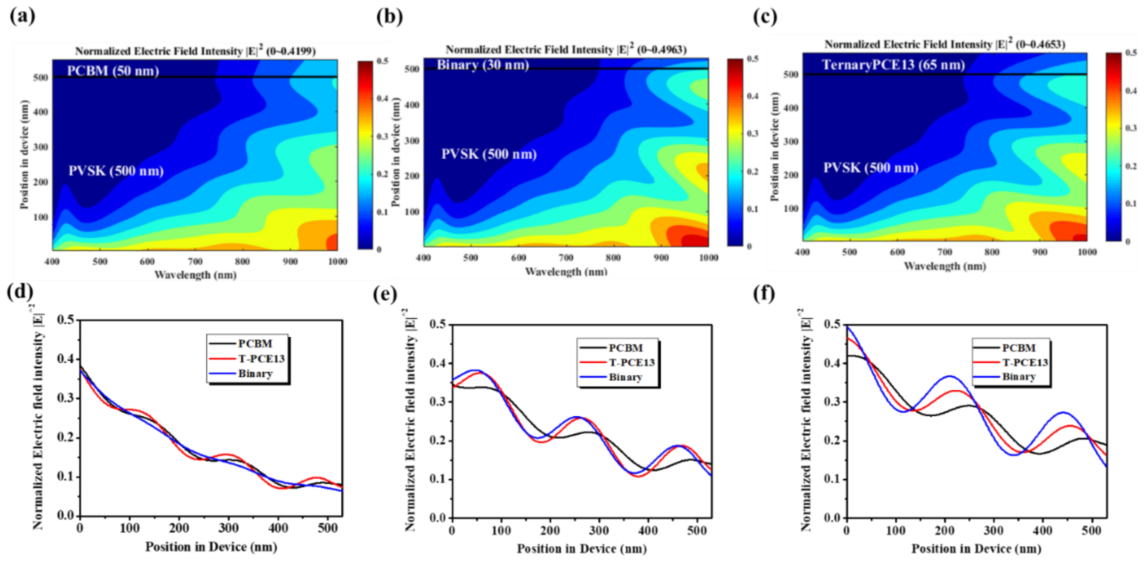

Figure S3. The simulated light intensity distribution in the studied devices using (a) a PCBM ETL, (b) a binary ETL, and (c) a T-PCE13 ETL. The optical response of (a) 800 nm, (b) 900 nm, and (c) 1000 nm in these devices.

Table S1. The estimated SCLC mobility of studied ETLs.

ETLs	μ_h (m^2/V s)	μ_e (m^2/V s)
**Binary**	9.39×10^{-5}	1.08×10^{-2}
**T-PCE12**	2.31×10^{-1}	3.68×10^{-2}
**T-PCE13**	3.01×10^{-1}	7.85×10^{-2}
**T-PCE13 w/ 1% DPE**	3.17×10^{-1}	9.41×10^{-2}

Table S2. The photovoltaic performance of studied $CH_3NH_3PbI_3$ PVSCs using a T-PCE13 ETLs processed with 1% DPE, 1% DIO, and 1% CN.

ETLs		Scan[a]	V_{oc} (V)	FF (%)	PCE (%)	J_{sc} (mA/cm^2)	
						J-V[b]	EQE[c]
Ternary-PCE13	1%DIO	f	0.95	53	8.8	17.3	17.5
		r	0.95	53	8.6	17.1	
	1%DPE	f	0.97	64	13.6	22.0	21.4
		r	0.98	64	13.8	21.9	
	1%CN	f	0.99	61	10.9	18.1	18.9
		r	0.99	58	10.5	18.3	

[a] f: measured under forward scan; r: measured under reverse scan.
[b] The current density derived from J-V curve at applied voltage to be 0.
[c] The integrated current density from 300 nm to 1000 nm.

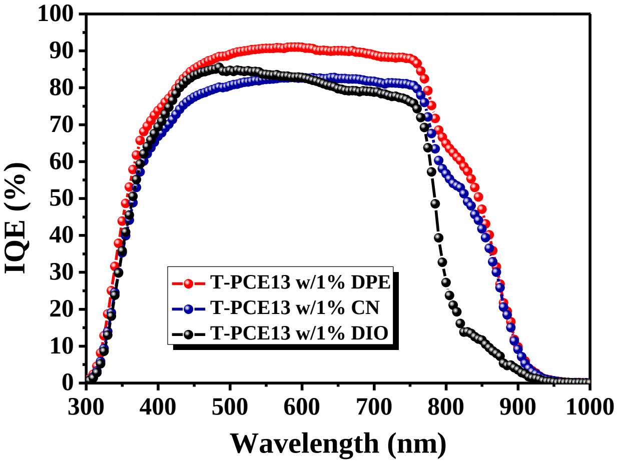

Figure S4. IQE spectra of the device using a T-PCE13 ETLs processed with 1% DPE, 1% DIO, and 1% CN.

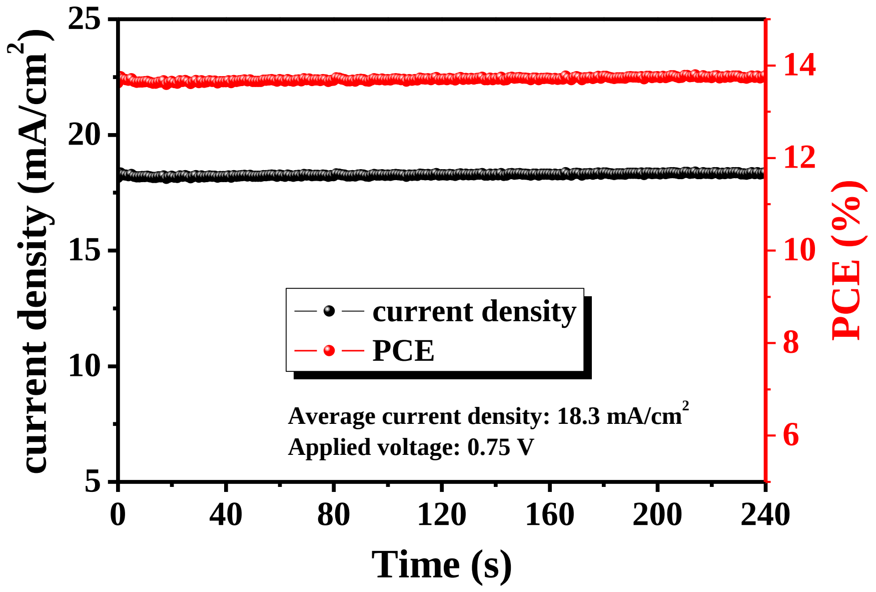

Figure S5. Steady-state current density and PCE for the $CH_3NH_3PbI_3$ device using a T-PCE13 w/ 1% DPE ETL.

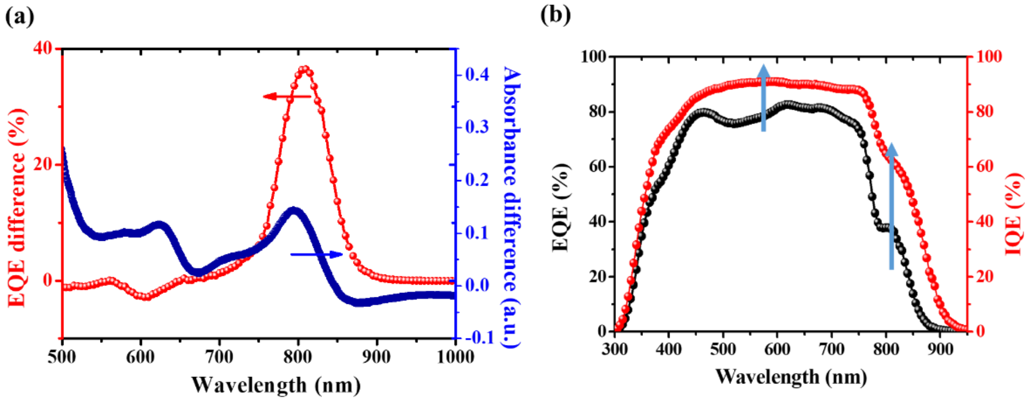

Figure S6. (a) The EQE difference and absorption difference between the devices using a bare PCBM ETL and using a T-PCE13 ETL processed with 1% DPE. (b) The comparison of the EQE and IQE spectra of the device using a T-PCE13 ETL processed with 1% DPE.

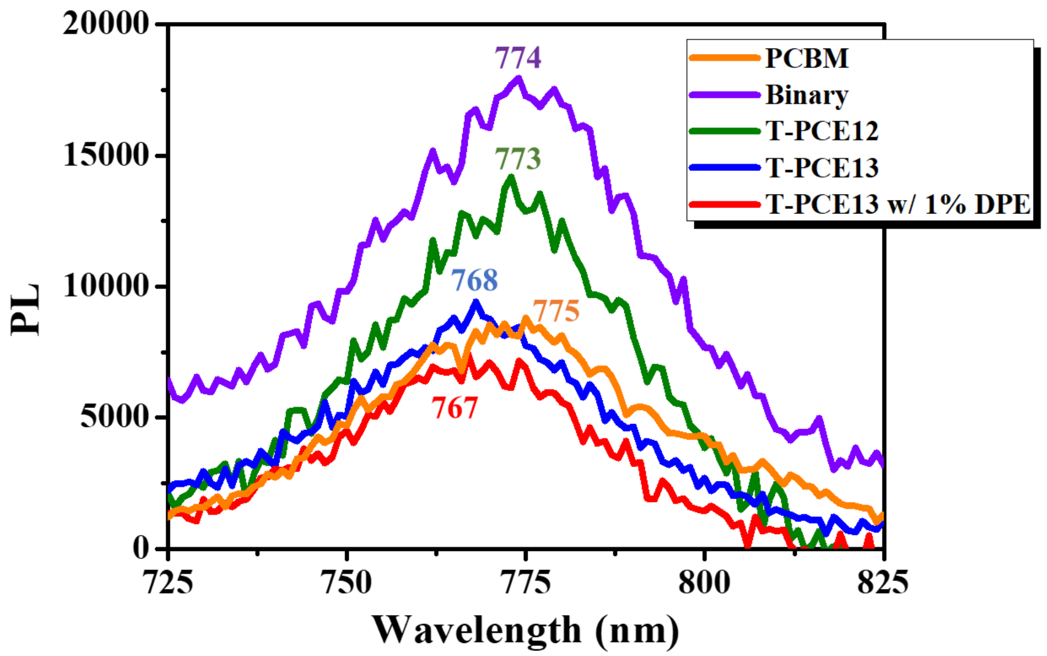

Figure S7. Amplification of the shifted PL shown in **Figure 3b**.

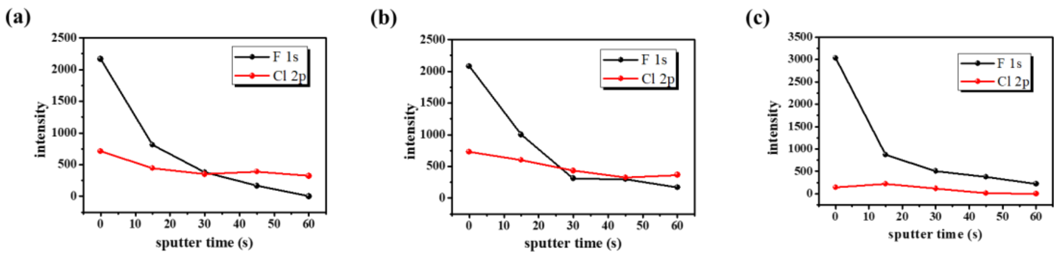

Figure S8. The XPS element intensity of F atom (related to PCE13) and Cl atom (related to BT-CIC) for depth profiling: (a) T-PCE13 ETL, (b) T-PCE13 w/ 1% DPE ETL, and (c) T-PCE13 w/ 1% DIO ETL.

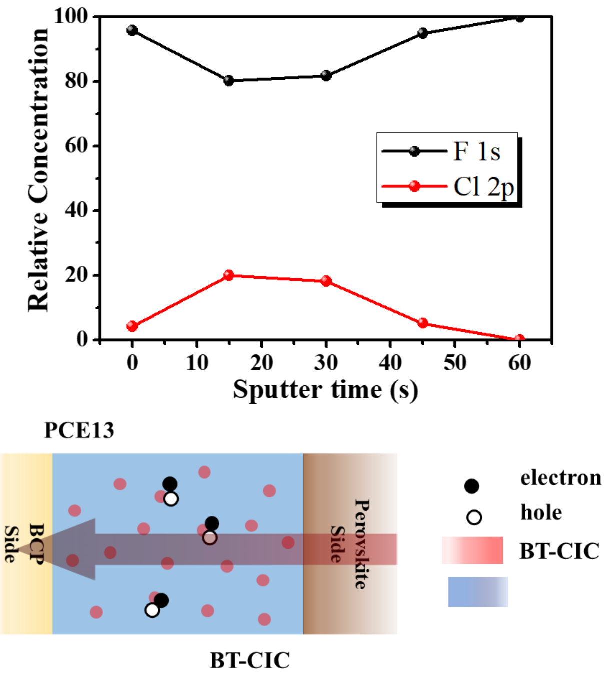

Figure S9. Normalized XPS element intensity of F atom (related to PCE13) and Cl atom (related to BT-CIC) for the relatively vertical concentration and associated illustration of distribution of components for T-PCE13 w/ 1% DIO ETL.

Table S3. TRPL results of a bare perovskite film and the bilayer perovskite/ETL films.

Employed ETLs	τ_1 (ns)	τ_1 weight (%)	τ_2 (ns)	τ_2 weight (%)	τ_{avg} (ns)
**None**	57.9	16.15	182.9	83.85	175.7
**PCBM**	2.4	32.66	21.5	67.34	20.5
**Binary**	3.3	26.25	26.1	73.75	25.1
**T-PCE13**	1.3	40.26	19.2	59.74	18.4
**T-PCE13 w/ 1% DPE**	1.3	36.73	16.5	63.27	15.8

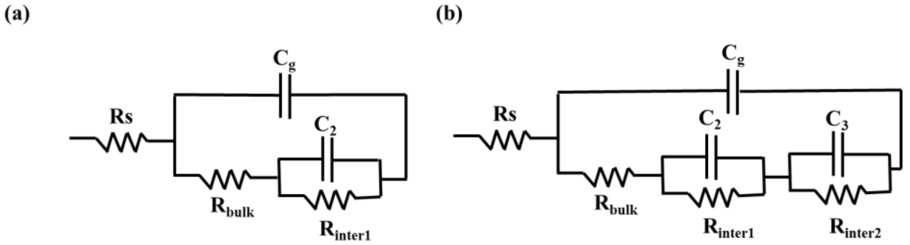

Figure S10. Equivalaent circuit for (a) the PCBM and T-PCE13 w/ 1% DPE as ETLs and (b) for the binary ETL consisting of BT-CIC:PCBM (8:12 mg/ml).

Table S4. The EIS fitting results of the devices using a bare PCBM ETL, a binary ETL, and a T-PCE13 w/ 1% DPE ETL.

ETLs	R_s (ohm)	R_{bulk} (ohm)	R_{inter1} (ohm)	R_{inter2} (ohm)
**PCBM**	39.24	173.3	139.6	--
**Binary**	56.74	183.4	76.9	81.45
**T-PCE13 w/ 1% DPE**	54.71	151.5	119.2	--

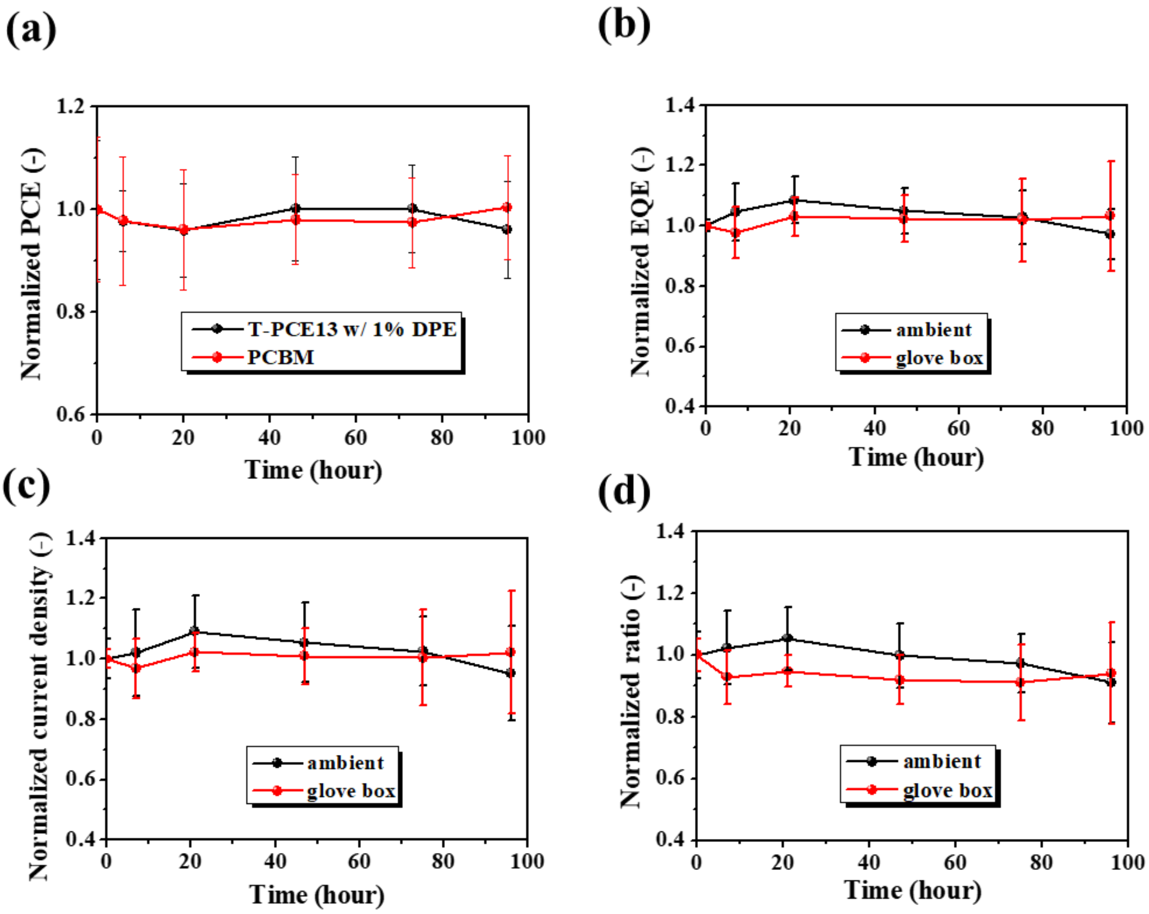

Figure S11. (a) Ambient stability test of the control device using a PCBM ETL and the device using a T-PCE13 w/ 1% DPE ETL. The stability of the NIR photo-response of the device using a T-PCE13 w/ 1% DPE ETL (stored in a glove box or in the ambient environment (room temperature and a relative humidity of 60 ± 5%) under dark without encapsulation): (b) the normalized EQE value at 800 nm, (c) the integrated current density above 800 nm, and (d) the contribution ratio of the integrated current density above 800 nm to the overall current density.
